# Reflections on the genetics-first approach to advancements in molecular genetic and neurobiological research on neurodevelopmental disorders

**DOI:** 10.1186/s11689-021-09371-4

**Published:** 2021-06-21

**Authors:** Anne B. Arnett, Tianyun Wang, Evan E. Eichler, Raphael A. Bernier

**Affiliations:** 1 Department of Psychiatry and Behavioral Sciences, University of Washington, CHDD, Box 357920, Seattle, WA 98195 USA; 2 Department of Psychiatry and Behavioral Medicine, Seattle Children’s Hospital, Seattle, WA USA; 3 Department of Genome Sciences, University of Washington, Seattle, WA USA; 4 Howard Hughes Medical Institute, University of Washington, Seattle, WA USA

**Keywords:** Neurodevelopmental disorders, Genetics-first, Molecular genetics, Neurobiology, Excitatory/inhibitory

## Abstract

**Background:**

Neurodevelopmental disorders (NDDs), including autism spectrum disorder (ASD) and intellectual disability (ID), are common diagnoses with highly heterogeneous phenotypes and etiology. The genetics-first approach to research on NDDs has led to the identification of hundreds of genes conferring risk for ASD, ID, and related symptoms.

**Main body:**

Although relatively few individuals with NDDs share likely gene-disruptive (LGD) mutations in the same gene, characterization of overlapping functions, protein networks, and temporospatial expression patterns among these genes has led to increased understanding of the neurobiological etiology of NDDs. This shift in focus away from single genes and toward broader gene–brain–behavior pathways has been accelerated by the development of publicly available transcriptomic databases, cell type-specific research methods, and sequencing of non-coding genomic regions.

**Conclusions:**

The genetics-first approach to research on NDDs has advanced the identification of critical protein function pathways and temporospatial expression patterns, expanding the impact of this research beyond individuals with single-gene mutations to the broader population of patients with NDDs.

## Background

Neurodevelopmental disorders (NDDs) encompass a wide range of cognitive, behavioral, motoric, and adaptive symptoms including autism spectrum disorder (ASD), intellectual disability (ID), and related symptoms. ASD is one of the common NDDs that begins in early childhood and is often associated with functional impairment throughout adulthood, affecting an estimated 1–2% of children worldwide [1, 2]. ASD is defined behaviorally by core deficits in social communication, repetitive behaviors, and restricted interests; yet, there is considerable variability in individual symptom presentation and developmental course [3, 4]. Likewise, ID is a NDD diagnosis given to individuals with a broad spectrum of impairment, ranging from mild difficulties with communication, problem solving, and adaptive functioning to profound difficulties with language, cognition, and independent living skills. Other common NDD symptoms include dysregulated attention, hyperactivity, and impulsivity characteristic of attention deficit hyperactivity disorder (ADHD); relative difficulty with phoneme awareness, i.e., dyslexia; impaired fine or gross motor coordination, i.e., dyspraxia; and language impairments. While these diagnoses commonly co-occur [5–8], the precise constellation of symptoms present for a given individual varies significantly across the NDD clinical population. This creates challenges for clinicians and families with respect to treatment planning, prognosis, and medical care. To parse the heterogeneity in NDDs, increasing attention has been paid to patients carrying ultra-rare or de novo likely gene-disruptive (LGD) mutations in genes that are known to be associated with those disorders.

Approximately 30% of ASD cases may be associated with rare or de novo variants (DNVs) within one of hundreds of NDD-associated genes or copy number variations (CNVs) [9–11]. Of these, a subset of “high-confidence” NDD genes have repeatedly been identified and have thus received the bulk of attention in human subject research. Genotype–phenotype correlation studies of high-confidence NDD genes, like *CHD8* [12, 13], *ADNP* [14–16], and *POGZ* [17, 18], have shown that individuals with LGD mutations in the same gene usually have common phenotypes, such as ASD, dysmorphic features, cognitive impairment, and medical conditions. Thus, progress in parsing the phenotypic heterogeneity of NDDs has already been made via characterization of phenotypic profiles associated with these genetic subtypes [19]. In a previous review, we concluded that this area of research has provided critical support to clinicians and families of affected individuals in the forms of diagnostic guidelines, clinical care, and socioemotional support. Moreover, the genetics-first approach has facilitated the development of precision medicine therapies into its early stages [20, 21]. In this current paper, we present a complementary discussion of advancements in understanding the molecular genetics and neurobiological basis of NDDs that have been achieved via the genetics-first approach. We focus our discussion on single-gene mutations, using examples from some of the most well-studied NDD genes, although we acknowledge that additional etiological complexities, including CNVs and gene–gene interactions, contribute to the phenotypic and genetic heterogeneity of NDDs. The LGD mutations reviewed herein have all been linked to ASD and/or ID; however, we emphasize that a crucial component of the genetics-first approach is that inclusion criteria are the common genetic etiologies, rather than shared phenotypic outcomes.

## Molecular genetic pathways associated with NDD

Following the initial discovery of high-confidence genes associated with ASD and related NDDs [22], Iossifov and colleagues [11] published groundbreaking evidence that, despite their vast number, many implicated genes converge on common molecular and functional pathways. These categories include genes involved in chromatin remodeling, fragile X mental retardation protein (FMRP) targets, postsynaptic density proteins, and genes expressed primarily during prenatal development. Since these original publications, the list of NDD-associated genes has grown, as has the complexity of their genetic interrelations and functional overlap. In the following section, we describe some of the molecular genetic pathways that are most commonly implicated in some of the high-confidence NDD genes. As examples, we prioritize and summarize 16 prevalent high-confidence NDD genes and their involvement in protein pathways in Table 1. These genes were selected for inclusion due to well-established evidence of their involvement in NDDs and the greatest number of DNVs reported in the extant literature. We acknowledge that NDD genes are involved in many other protein pathways, both known and unknown, and thus consider this discussion merely a starting point for future genetics-first research endeavors.

**Table 1:** Gene–protein network involvement of 16 prevalent high-confidence NDD genes

Gene	***Wnt/β***-catenin	FMRP	CHD8	TBR1
*ADNP*		[7]	ST [20]^a^	ST [23]
*ARID1B*	ST	[7]	ST [20]^a^	[23]
*ASH1L*			ST	
*ASXL3*			ST	
*CHD8*	ST [14]	ST [7]	[20]	ST
*DYRK1A*	ST	ST	ST	ST [23]
*FOXP1*	ST		ST	ST
*GRIN2B*	ST	ST [7]	ST	ST [23]
*MECP2*		ST	ST	ST
*POGZ*			ST [20]^a^	ST [23]
*PTEN*	ST [14]	ST	ST [20]	
*SCN2A*		ST [7]	ST [20]	ST [23]
*SHANK3*			[20]	
*KMT5B*			ST [20]^a^	
*SYNGAP1*		ST	ST [20]^a^	
*WDFY3*		[7]	ST	

Note: Numbers indicate sources (see References) that report gene involvement in each category. ST, STRING [24] interaction score of at least 0.30 with the protein as the central node. ^a^Gene had CHD8 binding sites but fold change was not statistically significant, as reported in [20]. *CHD2* was not included in the table as it was not involved in any of the reviewed protein pathways

Many high-confidence NDD genes are involved in the *Wnt-*signaling pathway, which activates *ϐ*-catenin and is essential for neuronal growth and proliferation, particularly during the embryonic phase of development [23, 25, 26]. Stessman and colleagues [27] reported preliminary findings that mutations to genes involved in the *Wnt*-signaling pathway (defined by a central node on *CTNNB1*, which encodes *ϐ*-catenin) resulted in two diametric phenotypes, one characterized by macrocephaly and the other by microcephaly. Although the molecular basis for this bimodal distribution remains unknown, gene–gene interactions and protein sub-networks are likely involved. In line with this, Chen and colleagues [28] subsequently reported that while *ϐ*-catenin signaling was elevated in the cerebral cortex of young *Pten*^*+/−*^ mice, leading to macrocephaly, cortical overgrowth could be suppressed with the introduction of an additional *Ctnnb1* heterozygous mutation. This study clearly demonstrated the importance of looking beyond single-gene variants to gene–gene interactions and protein subnetwork functionality to explain heterogeneity in NDDs.

One such gene–gene interaction network that clearly demonstrates the role of interactions among high-confidence NDD genes is that involving *CHD8*, which encodes chromodomain-helicase-DNA-binding protein 8, supporting chromatin remodeling and negatively regulating *Wnt* signaling [29]. The majority of individuals with a known LGD mutation in *CHD8* meet clinical criteria for ASD, while a significant proportion have intelligence in the average range [13], making *CHD8* one of the most ASD-specific single-gene subtypes. Interestingly, *CHD8* appears to regulate the expression of a number of other NDD-associated genes [12, 30], leading Beighley and colleagues [31] to investigate the phenotypic overlap between individuals with a *CHD8* mutation and those with a disruption to a gene that is regulated by the CHD8 protein (i.e., a “CHD8 Target”). Individuals carrying mutations in either *CHD8* or CHD8 Target groups had more severe social deficits, larger average head circumference, and a lower rate of seizures compared to individuals with LGD mutations in genes not regulated by CHD8. This phenotypic profile is highly consistent with the descriptions of “idiopathic” ASD, suggesting roles for CHD8 regulation and *Wnt* signaling in the broader NDD population [32].

Similarly, *TBR1* regulates the expression of many high-confidence NDD genes and is critical to early cortical development, particularly in the earliest stage of corticogenesis. *Tbr1* knockout mice showed dysregulated expression of other NDD genes, including reduced expression of *Arid1b*, *Ank2*, *Scn2a1*, and *Grin2b* and increased expression of *Adnp*, *Dyrk1a*, and *Pogz* [33]*.* In contrast, *Chd8* expression does not appear to be disrupted in *Tbr*^*−/−*^ mice, consistent with prior research that suggests *CHD8* and *TBR1* show at least partially independent expression patterns [34]. However, other studies have suggested that the coexpression levels of *TBR1* and *CHD8* vary by developmental stage [35], underscoring the complexity of gene–gene interactions over the lifetime.

Genes targeted by fragile X mental retardation protein (FMRP) [36] constitute another broad functional group associated with ASD [37, 38]. FMRP supports synaptic plasticity through the regulation of RNA transcription [36]. Trinucleotide repeat expansion of the X-linked *FMR1* gene leads to fragile X syndrome, which is highly comorbid with ASD as well as related NDDs [39]. *FMR1* is involved in common protein pathways with several NDD-associated LGD mutations identified [11]. Moreover, a genome-wide association study (GWAS) found high specificity of FMRP-targeted transcripts in common genetic variation associated with ASD [40]. They reported that common allelic variants associated with ASD were enriched for FMRP target transcripts, but not for gene sets related to mitochondrial, glial, oligodendrocyte, or astrocyte subcellular functioning. However, our own STRING analysis of a list of NDD-linked variants from a more recent GWAS involving more than 13,000 individuals with ASD [41] does not replicate this result. The progression from an investigation of the molecular basis of a rare, single-gene disorder (i.e., fragile X syndrome) to the etiology of a large, diverse phenotype (i.e., ASD) is the hallmark of the genetics-first approach to neurodevelopmental research.

Table 1 demonstrates striking overlap, across pathways (i.e., horizontally) as well as across genes (i.e., vertically), underscoring the complexity of common variant and LGD mutation expression associated with NDDs. To date, very few studies have aimed to parse the phenotypic heterogeneity of NDDs from the perspective of these pathways. A next step in genetics-first research on NDDs will involve quantification of the impact of an individual’s LGD mutations on their biological functioning, possibly through measurement of their response to pharmacological intervention, followed by estimation of weights and interactions of these gene networks on phenotypic outcomes, using path analysis and/or clustering algorithms.

## Neurobiological temporospatial and tissue-specific expression patterns

NDD-associated genes are expressed at high levels in the developing cortex during early to mid-fetal phases [34, 35, 42]. Consequently, disruptive mutations in these genes are expected to impact early, critical neuronal development, including neurogenesis and differentiation. Using large clinical cohorts from the Simons Simplex Collection (SSC) and University of Washington (UW), Trinh and colleagues [43] found poorer social, cognitive, and adaptive outcomes among individuals with mutations to genes expressed predominantly during the prenatal period, relative to those with mutations in genes primarily expressed during postnatal development. These findings were consistent regardless of whether the sample was restricted to individuals meeting clinical criteria for ASD. Willsey and colleagues [35] investigated NDD-associated genetic expression at the cellular level and found that networks of high-confidence NDD genes (e.g., *CHD8*, *POGZ*, *DYRK1A*, and *TBR1*) were highly expressed in early to mid-fetal brains, particularly in deep-layer cortical projection neurons. These results potentially narrow the pathogenesis of NDDs to malfunctions in the first neural circuits of the developing fetal cortex. If very early synaptic dysfunction indeed constitutes the first step in the neurobiological etiology of ASD and related NDDs, this research also underscores the vast opportunity for individual differences in common genetic variation and environmental experiences to contribute to phenotypic heterogeneity across the lifespan.

In contrast, Hormozdiari et al. [44] reported on a set of high-confidence NDD genes that were differentially expressed during postnatal cortical development and enriched for involvement in long-term potentiation and synaptic plasticity, including *GRIN2B*, *SYNGAP1*, *STXBP1*, and others. DNVs in this gene set were associated with lower cognitive functioning and higher risk of epilepsy, but not ASD without cognitive impairment. Evidence to support a role of postnatally expressed genes in ASD can be found in the high rate of macrocephaly that occurs as a result of rapid growth in early development in this clinical population [32, 45]. Genetic syndromes caused by germline mutations in *PTEN* have been associated with both congenital and developmental macrocephaly [46] and ASD. *PTEN* acts as a tumor suppressor and is expressed widely throughout the body. In the brain, *PTEN* is differentially expressed in the cerebellum during postnatal development [47]. Haploinsufficient *Pten* mice show neuronal overgrowth and develop macrocephaly, social atypicalities, anxiety, and learning deficits similar to those seen in humans with ASD [48]. Butler and colleagues [49] identified *PTEN* mutations in 3/18 patients presenting with both ASD and macrocephaly. Additional research has confirmed that humans with *PTEN* mutations have neuronal overgrowth as well as white matter atypicalities on imaging [50]. While the exact role of *PTEN* in regulating neuronal growth appears to vary by cell type and developmental stage [28], this research contributes to our understanding of atypical neuroimaging and electrophysiology findings among children with “idiopathic” ASD, particularly those presenting with macrocephaly. Importantly, distinctions between *PTEN* and “idiopathic” ASD phenotypes are likely as informative as their overlap. For example, while macrocephaly is often detected at or before birth among children with *PTEN* mutations, macrocephaly among non-LGD ASD individuals typically develops in early or middle childhood [32]. This would suggest different genetic expression patterns between the two groups and may even support different protein networks. As large-scale transcriptomic databases are further developed to comprehensively characterize healthy genetic expression from conception through adulthood, we anticipate a greater understanding of the functional impact of LGD mutations in NDD-associated genes as well as common variants across development.

**Fig. 1:**
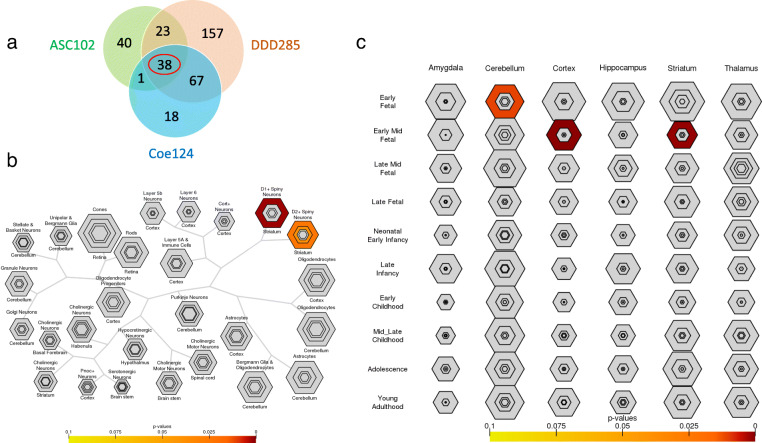
Temporospatial expression patterns of top NDD genes. **a** Overlap of three main significant gene lists, ASC102 [57], Coe124 [42], and DDD285 [58], suggesting 38 top risk NDD genes. **b** Using CSEA tool (http://genetics.wustl.edu/jdlab/csea-tool-2/) across cell types with 38 top genes showing enriched expression in D1+ (p-value = 3.1e−04, overlapped genes: *PPP2R5D*, *KCNQ3*, *GRIN2B*, *SHANK3*, *FOXP1*, *MYT1L*, *MED13L*) and D2+ (p = 0.006, overlapped genes: *KCNQ3*, *SHANK3*, *FOXP1*, *MYT1L*, *MED13L*) spiny neurons. **c** CSEA across brain regions and development stages with 38 top genes showing enriched expression in the cerebellum at early fetal stage (p-value = 0.002, overlapped genes: *ADNP*, *FOXP1*, *BCL11A*, *KDM5B*, *ARID1B*, *ASXL3*, *DNMT3A*), in the cortex at early mid-fetal stage (p-value = 1.6e−07, overlapped genes: *TCF4*, *GNAI1*, *ADNP*, *KDM5B*, *KMT5B*, *MYT1L*, *TCF20*, *ASXL3*, *CREBBP*, *GRIN2B*, *BCL11A*, *MED13L*), and in the striatum at early mid-fetal stage (p-value = 1.3e−04, overlapped genes: *FOXP1*, *MYT1L*, *KDM5B*, *TCF20*, *ARID1B*, *BCL11A*, *DNMT3A*)

Cell type-specific expression analysis (CSEA), through which genes are categorized according to the neural tissues in which they are expressed, presents another meaningful approach to parsing heterogeneity in NDDs [51]. Many NDD-associated genes and CNVs are expressed in the striatum, particularly dopamine receptor expressing D1^+^ and D2^+^ medium spiny neurons [42, 52–54], which are involved in excitatory and inhibitory dopaminergic pathways. Interestingly, this enrichment is found even among missense mutations and CNVs to non-coding regions of the genome [52]. Striatal dysfunction has been linked to motoric and sensory symptoms of autism [55] and has been associated with stereotyped motor movements in male, but not female mice, mirroring the sex distribution of ASD in humans [56]. Despite this converging research, it is important to note that NDD genes are expressed widely across brain regions and tissues [42]. Enhanced enrichment for NDD genes in D1+ and D2+ spiny neurons may be due in part to the reduced expression-level complexity for this particular neural tissue. Thus, while cell type-specific methodology holds promise for parsing the neurobiological and genetic heterogeneity of NDDs, this research is in its infancy and will be strengthened by investigation of gene–tissue associations that predict unique cognitive and behavioral outcomes [42] (Fig. 1).

## Clustering of de novo variants

**Fig. 2:**
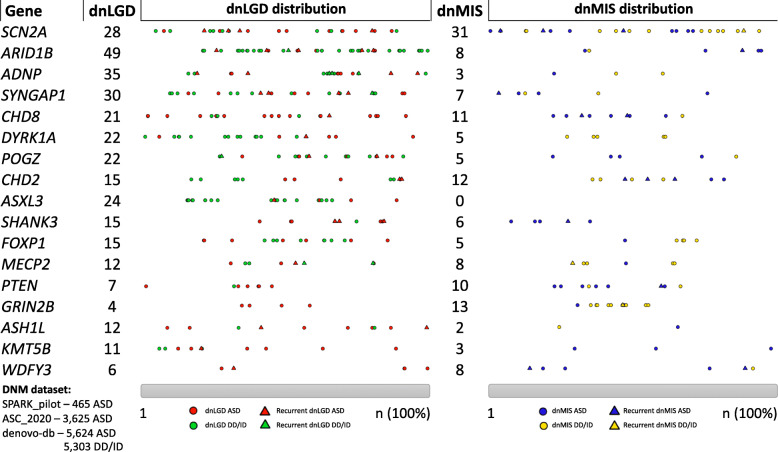
Distribution of dnLGD and dnMIS for 17 top NDD risk genes. DNVs were collected from unique samples of 10,927 NDD (5624 ASD and 5303 DD/ID) trios in denovo-db v1.5 [60], 465 ASD trios from the SPARK pilot study [62], and 3625 ASD trios from ASC study [54]. Samples that overlapped across studies were only counted once. The number of dnLGD and dnMIS is listed next to each gene, and the relative positioning of each variant was scaled in 1 to 100% based on the size of each gene

Identification of DNVs associated with NDDs has brought a group of genes to the forefront of neuroscientific research [11, 42, 59–61]. As a result, we have gained substantial insight into the discrete neurobiological functions of many high-confidence NDD genes, which is critical for the development of novel, genetically informed therapies. In contrast to their overlap with broad genetic pathways, the specific neurobiological function of individual NDD genes is highly variable. Moreover, a clear dispersion and clustering of the de novo LGD (dnLGD) and missense (dnMIS) variants across these genes is shown in Fig. 2, each of which could have critically distinct effects. In this section, we will describe genes with specific patterns of location for DNVs across the gene body.

De novo disruptions to *ARID1B* (AT-rich interaction domain 1B) are associated with ASD, Coffin-Siris syndrome, agenesis of the corpus callosum, and short stature, possibly mediated by dysregulation of the *Wnt/β*-catenin pathway [63]. Figure 2 shows that *ARID1B* is highly intolerant to mutations, with dnLGDs spanning the gene, in contrast to very few dnMISs identified from NDD patients. This finding highlights the importance of the ARID1B protein to cortical development and simultaneously introduces the potential for significant heterogeneity in phenotypic outcomes for patients carrying dnLGDs in *ARID1B*. In our own sample of eight individuals with a dnLGD in *ARID1B* from SSC and UW, cognitive and adaptive abilities ranged from severely impaired to the broadly average range; likewise, ASD symptom profiles ranged from significant social impairments with relatively few repetitive and restricted behaviors to the opposite presentation to severe symptoms in both domains.

In contrast, Fig. 2 demonstrates a high degree of clustering of dnLGDs in *ADNP*. The majority of documented mutations in *ADNP* are located on the fifth exon [64]. This region encodes the neuroprotective NAPVSIPQ (NAP) peptide, which is known to facilitate tau binding to microtubules, and is also strongly implicated in Alzheimer’s disease [15, 65]. Individuals with mutations in *ADNP* show a range of cognitive and behavioral impairments, but striking consistency of motor difficulties and low language acquisition [16, 66]. The specificity of these outcomes suggests direct associations between molecular genetics and phenotypic differences that could inform understanding of healthy brain development, as well as treatment targets.

Mutations in *SCN2A*, which may occur in up to 1% of ASD cases [67], impact the expression of a voltage-gated sodium channel, Na_V_1.2. Na_V_1.2 is found on GABAergic neurons and facilitates action potentials along the axon and also mediates the backpropagation of the action potential to the soma, a function thought to be critical for synaptic plasticity [68]. *SCN2A* variants differentially impact the excitability of the neuron depending on the location of the variant and corresponding Na_V_1.2 channel disruption. LGD mutations to *SCN2A* result in dampened Na_V_1.2 channel function, limiting or interrupting the action potential [69], and are commonly associated with ASD [69]. In contrast, gain-of-function *SCN2A* variants, which confer increased neuronal excitability, are associated with infantile-onset seizures [70]. Figure 2 shows that nearly equal proportions of dnLGD and dnMIS are characterized in SCN2A, but we see four dnLGDs are recurrently mutated in ASD patients, indicating the importance of these locations in ASD. Consistent with this, the direction of functional impact on Na_V_1.2 varies considerably depending on where the mutation is expressed on the protein [69]. However, both gain- and loss-of-function DNVs in *SCN2A* nearly always result in severe cognitive and behavioral impairment. This genotype aligns with the recent conceptualization of NDDs as disorders of atypical balance of neuronal excitation/inhibition, as well as conflicting reports regarding whether ASD, in particular, is characterized by increased excitation or inhibition, or both [71, 72].

## Advancements in precision medicine care

Many LGD mutations present clear targets for genomic and pharmacological therapies. For example, the specificity of the genotype–phenotype associations among loss-of-function versus gain-of-function variants in *SCN2A* suggests targeted modulation of Na_v_1.2 may be effective [20]. However, the development of genetics-based therapeutics for ASD and related NDDs is still in its infancy. *Fmr1* insufficient and 16p11.2 deletion mouse models have suggested modulation of excitatory and inhibitory neurotransmitters may be effective. *Fmr1* insufficient mice treated with mGluR5 antagonists demonstrated promising improvements in cognition, growth, and seizures [73]. The GABA_B_ receptor agonist arbaclofen improved cognitive and behavioral deficits and showed corresponding improvement in electrophysiological signals among 16p11.2 deletion mice [74]. Yet, randomized clinical trials of mGluR5 antagonists and arbaclofen in humans with ASD and/or fragile X syndrome have been less successful [75]. Although a small, open-label study suggested behavioral benefits of lithium among individuals with fragile X syndrome [76], a larger randomized control study of mGluR5 antagonist in this population did not show any improvement over placebo [77]. Arbaclofen clinical trials likewise failed to demonstrate improvement over placebo on primary social outcomes, but did report secondary benefits, such as reduced irritability and higher parent-reported adaptive social skills [21, 78]. Additional clinical trials with human subjects are underway in Europe and the USA.

The discovery of deficient NAP peptide associated with *ADNP* mutations has led to the pre-clinical development of a pharmaceutical version of NAP, CP201 [15]. In heterozygous *Adnp-*deficient mice, administration of CP201 led to increased dendritic spine density, increased vocalization, and normalization of motoric functions [79]. Similarly, pre-clinical studies have demonstrated that low doses of ketamine increase *ADNP* gene expression [80, 81], paving the way for a clinical trial of ketamine to treat *ADNP* mutation patients (currently in phase 2; ClinicalTrials.gov identifier: NCT04388774). The impact of this line of research has the potential to expand beyond *ADNP* mutations to treatment of Alzheimer’s disease, given the dysfunctional tau binding observed in both patients [15].

Categorization of NDD-associated genes according to temporospatial expression patterns may also inform the development of precision medicine care [43]. Postnatal development is associated with experience-dependent learning and sensitive periods of cortical plasticity. The effects of gene-therapy treatments and even behavioral interventions could be maximized by a precision medicine approach to delivering these interventions at sensitive developmental periods [82]; timing of interventions may be even more critical among individuals with LGD mutations to postnatally expressed genes. In contrast, individuals with prenatally expressed LGD mutations may be candidates for in utero gene or stem cell therapies, although the development of these approaches is in its infancy and presents with many technical, safety, and ethical challenges [83, 84].

## Limitations and future directions

Human subjects’ research on rare, de novo genetic mutations is inherently limited by low statistical power. Moreover, recruitment is likely biased toward the over-inclusion of individuals with a high degree of functional and behavioral impairment that prompted genetic testing in the clinical setting. Access to large-scale, population-based genomic databases will undoubtedly widen the spectrum of known phenotypes associated with high-confidence NDD genes in future research. Simultaneously, the current review highlights the potential to increase statistical power by characterizing individuals according to broader protein networks and gene expression properties, rather than single-gene disruptions. The next step in this research will be to investigate the impacts of intra- and inter-network interactions among de novo and common variants.

While this line of inquiry appropriately mirrors the complexity of the human genome, we acknowledge that this research will be extremely challenging. Additionally, we recognize that attempts to identify common neurobiological etiology, such as E/I imbalance or shared protein pathways, are somewhat antithetical to the genetics-first emphasis on individual heterogeneity in NDDs. Nonetheless, we believe this line of work is critical to furthering our understanding of individual LGD mutation, as cross-LGD comparisons increase statistical power, contribute to the broader understanding of human neurobiology, and lead to the discovery of both shared and distinct gene–brain–behavior pathways.

The aims of genetics-first research on NDDs are multiplicative, but in our opinion primarily address the basic science need to better characterize genetic–neurobiological associations across development, and the clinical science need to develop precision medicine care for individuals with NDDs. In the spirit of parsing neurodevelopmental heterogeneity from multiple angles, we propose that future research could investigate the genetic and neurobiological profiles of individuals who show positive, neutral, and adverse responses to novel therapeutics, such as GABA and glutamate modulators. The potential for individual differences in medication response to inform understanding of neurobiological heterogeneity has been demonstrated in research on other neuropsychiatric disorders, such as attention deficit hyperactivity disorder [85], and may provide additional clues about individual differences in brain–behavior associations with NDD genes.

## Conclusions

Genetics-first research on NDDs has gained momentum over the past decade, with the advent of improved genomic sequencing methods, global human subjects’ recruitment efforts, and development of healthy and neuropsychiatric transcriptomic databases. Characterization of high-confidence NDD genes into broader categories of protein function and temporospatial expression patterns furthers the understanding of genetic–neurobiological pathways and expands the impact of this research beyond individuals with single-gene mutations to the broader NDD population. Moreover, research funding may be easier to access as this research increasingly shifts from bench to bedside, with a focus on deriving practical implications for the development of precision medicine care for this diverse clinical population.

## Data Availability

Not applicable.

## Abbreviations

NDDs: Neurodevelopmental disorders
ASD: Autism spectrum disorder
GWAS: Genome-wide association study
CNVs: Copy number variations
CSEA: Cell type-specific expression analysis
FMRP: Fragile X mental retardation protein
LGD: Likely gene-disruptive
dnLGD: De novo LGD variant
dnMIS: De novo missense variant
DNV: De novo variant
